# Supplementary material for: Recurrent Processing Drives Perceptual Plasticity
Source: Curr Biol. 2020 Nov 2;30(21):4177–4187.e4. doi: 10.1016/j.cub.2020.08.016 (PMC7658806; doi:10.1016/j.cub.2020.08.016)
Supplement: Document S1. Figures S1–S4 [file mmc1.pdf]

Current Biology, Volume *30*

Supplemental Information

Recurrent Processing Drives Perceptual Plasticity

Ke Jia, Elisa Zamboni, Valentin Kemper, Catarina Rua, Nuno Reis Goncalves, Adrian Ka Tsun Ng, Christopher T. Rodgers, Guy Williams, Rainer Goebel, and Zoe Kourtzi

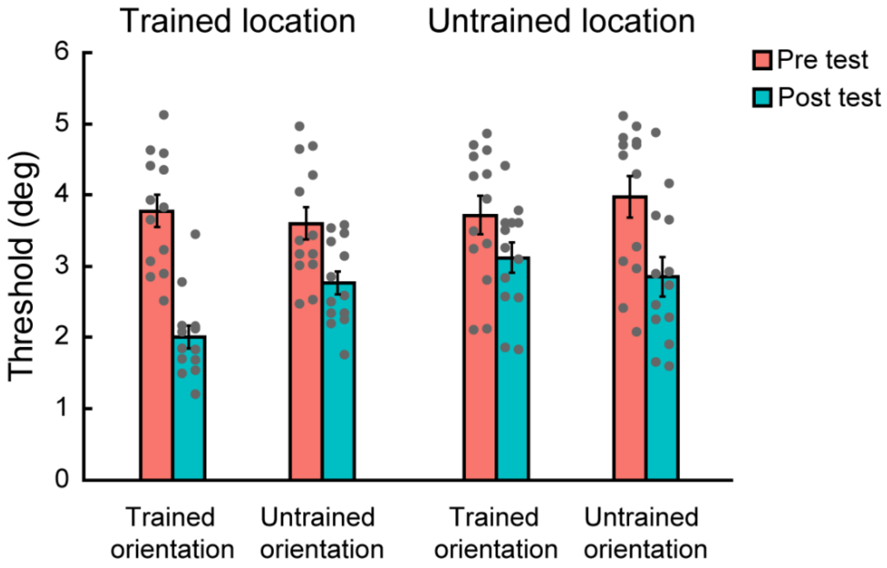

Figure S1. Behavioral results. Related to Figure 2D.

Mean performance across participants before and after training (pre-, post-test) at ~79.4% threshold for the trained and untrained orientations presented at the trained and untrained locations. Error bars indicate standard error of the mean across participants. Gray dots indicate the threshold of each participant. We observed learning specificity for the trained orientation at the trained location, as indicated by a significant orientation × location × session interaction (repeated measures ANOVA, $F(1,12) = 11.858$, $p = 0.005$) and a significant orientation × session interaction ($F(1,12) = 21.551$, $p = 0.001$) at the trained, but not the untrained ($F(1,12) = 3.093$, $p = 0.104$) location. Post-hoc comparisons at the trained location showed significantly lower threshold for the trained than the untrained orientation after ($t(12) = -5.208$, $p < 0.001$), but not before ($t(12) = 1.264$, $p = 0.230$) training. In contrast, no significant differences between the trained and the untrained orientations were observed at the untrained location (pre-test session: $t(12) = -1.203$, $p = 0.252$, post-test session: $t(12) = 1.066$, $p = 0.308$).

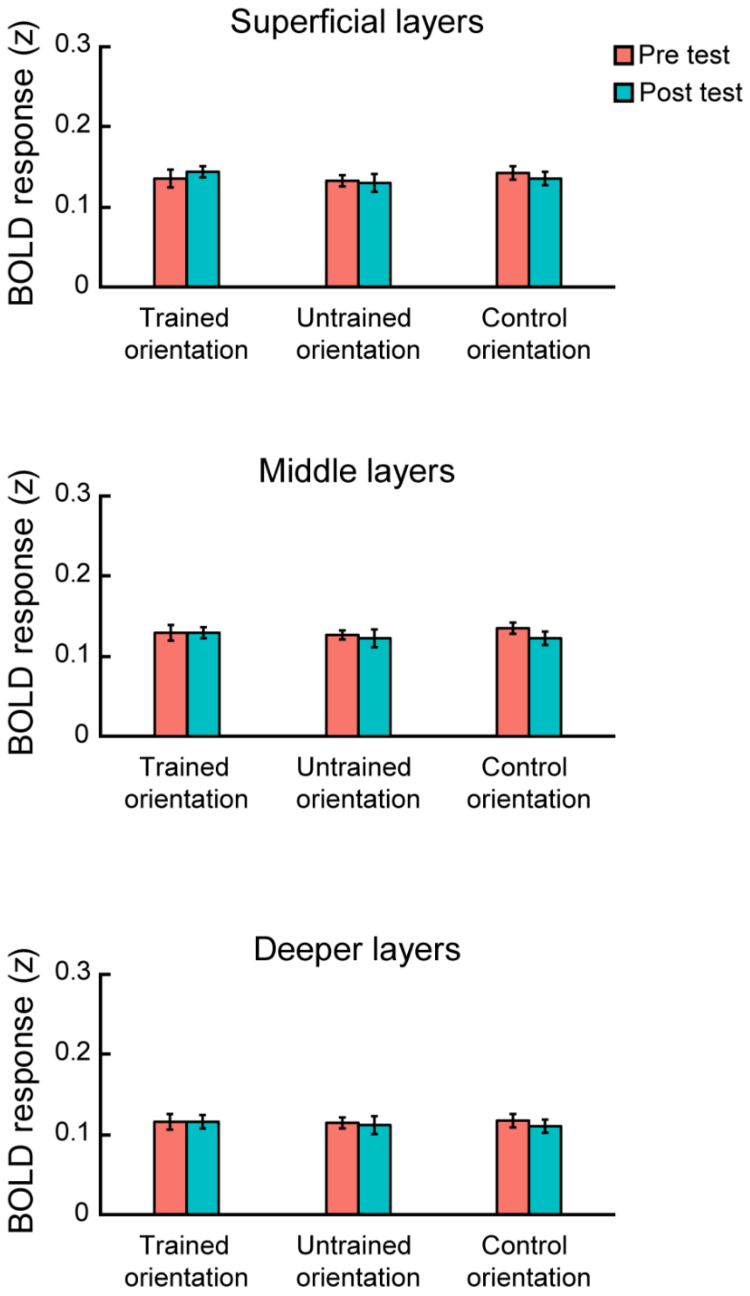

Figure S2. Univariate fMRI analysis. Related to Figure 4.

Mean normalized BOLD before and after training for the trained, untrained and control orientations presented at the trained location across V1 layers. Error bars indicate standard error of the mean across participants.

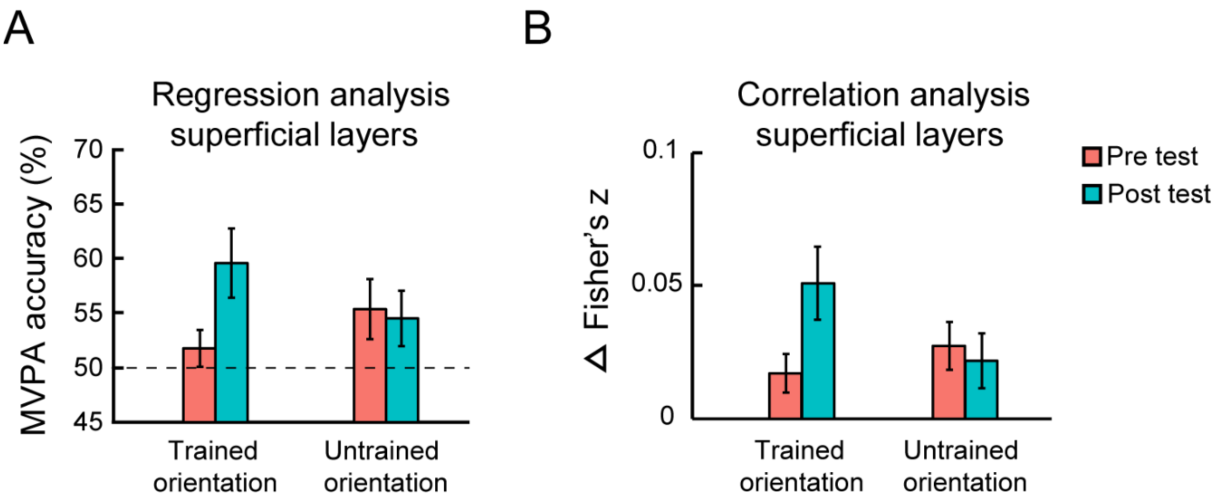

Figure S3. Control analyses. Related to Figure 4.

(A) MVPA accuracy before and after training for the trained and untrained orientations presented at the trained location in superficial V1 layers after regressing out the signal from the adjacent voxels in middle layers. Dotted line indicates MVPA accuracy at 50% chance. (B) Correlation-based pattern analysis. Correlation differences (correlation of mean normalized BOLD across voxels for the same orientation minus normalized BOLD for different orientations) for the trained and untrained orientations presented at the trained location in superficial V1 layers. Error bars indicate standard error of the mean across participants.

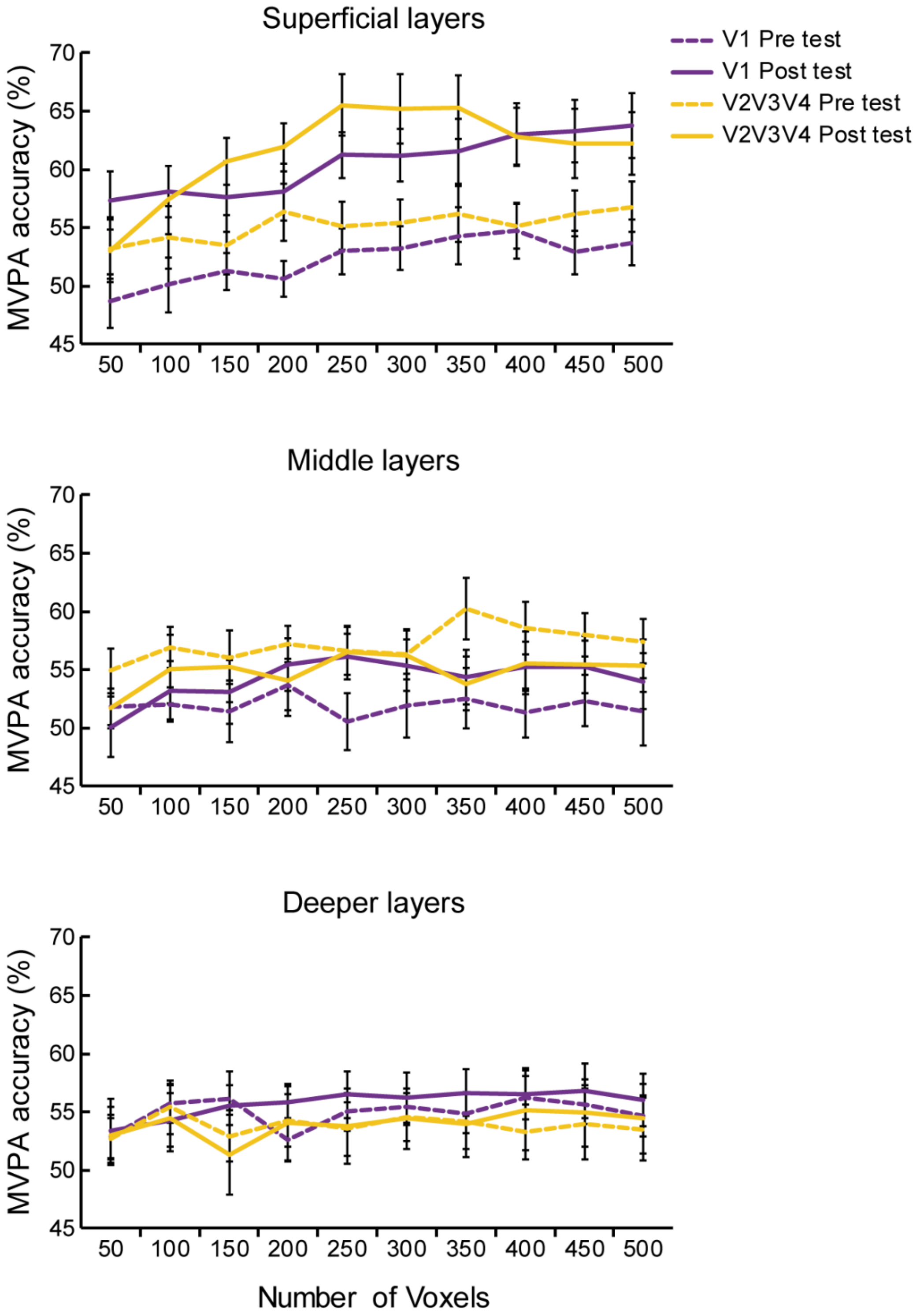

Figure S4. MVPA before and after training across visual areas. Related to Figure 5.

MVPA accuracy before and after training for different voxel patterns (from 100-500 voxels) for the trained orientation presented at the trained location across layers of V1, V2, V3, V4. We observed similar learning-

dependent changes in superficial layers across visual areas. Two-way repeated measures ANOVAs (ROI × session) showed a significant main effect of session for pattern size of 200 ($F(1,12) = 7.159$, $p = 0.020$), 300 ($F(1,12) = 7.751$, $p = 0.017$), 400 ($F(1,12) = 6.006$, $p = 0.031$) voxels, and a trend for pattern size of 100 ($F(1,12) = 3.743$, $p = 0.077$) and 500 ($F(1,12) = 4.587$, $p = 0.053$) voxels. We did not observe any significant ROI × session interaction (all *ps* > 0.269). Further, we did not observe any significant differences in MVPA accuracy before vs. after training in middle nor deeper layers (all *ps* > 0.539). Error bars indicate standard error of the mean across participants.
